# Comparing cardiac troponin levels using sevoflurane and isoflurane in patients undergoing cardiac surgery: a systematic review and meta-analysis

**DOI:** 10.34172/jcvtr.2020.01

**Published:** 2020-02-12

**Authors:** Hossein Hosseinifard, Nashmil Ghadimi, Sara Kaveh, Hossein Shabaninejad, Alaadine Lijassi, Rasoul Azarfarin

**Affiliations:** ^1^Biostatistics, School of Health Management and Information Sciences, Iran University of Medical Sciences, Tehran, Iran; ^2^Health Technology Assessment, School of Health Management and Information Sciences, Iran University of Medical Sciences, Tehran, Iran; ^3^Department of Health Services Management, School of Health Management and Information Sciences, Iran University of Medical Sciences, Tehran, Iran; ^4^Population Health Sciences Institute, Newcastle University, Newcastle, United Kingdom; ^5^Faculty of Medicine and Pharmacy of Rabat, Mohammed V University of Rabat, Rabat, Morocco; ^6^Echocardiography Research Center, Rajaie Cardiovascular Medical and Research Center, Iran University of Medical Sciences, Tehran, Iran

**Keywords:** Troponin, Sevoflurane, Isoflurane, Cardiac Surgery

## Abstract

***Introduction:*** Cardiac troponin is one of the heart biomarkers and its high levels correlates with a high risk of cardiomyocytes damage. This study aimed to compare sevoflurane and isoflurane effect on troponin levels in patients undergoing cardiac surgery.

*** Methods: *** We systematically searched for RCTs which had been published in Cochrane library, PubMed, Web of science, CRD, Scopus, and Google Scholar by the end of February 30th, 2019. The quality of articles was evaluated with the Cochrane checklist. GRADE was used for quality of evidence for this meta-analysis. Meta-analysis was done based on random or fixed effect model.

*** Results:*** Five studies with total of 190 (sevoflurane) and 191 (isoflurane) patients were included. The results showed that pooled mean difference of troponin levels between the two groups was significant at ICU admission time and 24 hours after entering. The comparison of troponin level changes between the two groups (baseline = at time ICU) in 24 and 48 hours after ICU admission was significant.

***Conclusion:*** This meta-analysis showed that blood troponin levels were significantly lower at the time of arrival in ICU with isoflurane and after 24 hours with sevoflurane. Generally, given the small mean difference between isoflurane and sevoflurane, it seems that none of the medications has a negative effect on the cardiac troponin level.

## Introduction

Cardiovascular disease is the most common cause of death. One of the approaches considered for the treatment of patients with this disease is cardiac surgery. Patients undergoing heart surgery are usually at risk of developing myocardial damage.^1-3^ There are several ways to evaluate cardiac damage and monitor the status of heart during surgery. Cardiac enzymes such as troponin T and I are proprietary biomarkers which are not detectable in healthy people.^4-6^ Troponins are regulatory proteins present in actin filaments that contribute to the regulation of the contraction of the heart cells. When a patient suffers from heart attack, the amount of these enzymes changes and increases. Therefore, troponins are considered as high sensitivity markers to measure patients’ cardiac status.^6-9^

Today, the use of volatiles anesthetics such as isoflurane and sevoflurane has expanded in many surgical procedures, including heart surgery.^10,11^ These two volatile anesthetics have different effects on cardiac function.^12^ However, sevoflurane is less soluble than isoflurane.^13^ Several animal studies have shown that volatiles anesthesia has a protective effect on myocardial infarction.^14-17^ Human studies also indicate that these drugs have the potential to provide adequate anesthetic depth. Moreover, these drugs have protective effects on myocardial infarction. Furthermore, research studies, which have examined the amount of troponin enzyme using isoflurane and sevoflurane judges, have shown different results.^18-21^ Troponin often presents a unique prognosis of patient conditions in the ICU and 24 hours after the operation.^22^

## Aim of the Review

In this regard, the aim of this study was to compare the effect of isoflurane and sevoflurane on troponin levels in patients undergoing cardiac surgery in a systematic review and meta-analysis. The results of this study would help clinicians obtain evidence based on clinical evidence and select the best inhalational anesthetic agent.

## Methods

This was a systematic review and meta-analysis study that utilized randomized controlled trials (RCTs) to compare the effects of sevoflurane and isoflurane on troponin levels in patients with cardiac surgery, without any time constraints and based on Preferred Reporting Items for Systematic Reviews and Meta-Analyses (PRISMA) guidelines.^23^

### Systematic literature search

We systematically searched for RCTs published in Cochrane library, PubMed, Web of science, CRD, Scopus and Google Scholar databases until February 30, 2019. Keywords include ‘Sevoflurane’, ‘Isoflurane’, ‘Anesthesia’, Coronary artery bypass surgery’, ‘Heart surgery’, ‘randomized controlled trials’, and corresponding MeSH terms. The reference list of selected studies was searched manually. Hand-searching and studies presented at conferences were also searched (literature searches, online Resource 1, Supplementary file 1).

### Selection of included studies

Studies met the following inclusion criteria in our meta-analysis if: (1) they studied patients who underwent cardiac surgery and reported the results; (2) patients had to have at least one troponin measurement during cardiac surgery; (3) patients undergoing cardiac surgery used sevoflurane and isoflurane in their anesthesia; and (4) the study was a prospective, randomized, controlled clinical trial. Exclusion criteria were (1) comparison of other anesthetic drugs; (2) other kinds of surgeries; (3) studies that did not report on specific outcomes; (4) studies that had incomplete data about the mean and standard deviation of the desired outcomes; and (5) animal studies.

### Study selection and data extraction

The Endnote X5 resource management software was used to organize, study the titles and abstracts, and identify duplicates. After removing duplicate articles, the abstracts and the full texts of the articles were examined and those which met eligibility criteria were selected. The extracted data were transferred into a table. The extracted data include: First author, year of publication, the country in which the study had been performed, type of interventions, the number of subjects in the control and intervention group, type of study, mean and standard deviation of troponin levels, and finally (cTnT and/or cTnI(.

### Study quality and risk of bias

The quality evaluation of the studies was done using the Cochrane Collaboration’s tool^24^ in the RevMan 5.3 software. In assessing the quality of the studies, the domains considered included (i) random sequence generation (selection bias), (ii) allocation concealment (selection bias), (iii) blinding of participants and personnel (performance bias), (iv)blinding of outcome assessment (detection bias), (v) incomplete outcome data (attrition bias), (vii) selective reporting (reporting bias), and other bias.

During all stages of study selection, data extraction and quality assessment of studies were done by two of the researchers independently. Any disagreement between the researchers was discussed with a third reviewer and the consensus was gained

### Quality of the evidence

A structured and transparent GRADE approach study was performed (Grading of Recommendations Assessment, Development and Evaluation).^25^ In the present study, this approach measured the strength of the evidence obtained from primary and secondary outcomes as well as subgroups from low to high in order to study the strength of GRADE evidence, heterogeneity between studies was considered by Indirectness, Imprecision, Publication bias and Power of relation.

### Statistical analysis

The sample size, mean troponin level, and standard deviation were extracted from the studies before the induction time, at the time of being in ICU, and 6, 12, 24 and 48 hours after entering the ICU. The difference in mean troponin levels at the above mentioned time points was calculated in both groups of sevoflurane and isoflurane compared to before the induction levels. Finally, using meta-analysis based on random or fixed effect model, the mean differences in troponin levels in the anesthetics group of sevoflurane and isoflurane were combined. The heterogeneity between studies was investigated using chi-square q-test and I^2^ statistics which expresses the percentage of variation between studies. I^2^ values less than 25% indicated low heterogeneity, I^2^ values between 25% and 75% was regarded average heterogeneity, and over 75%, heterogeneity was considered high. In case of significant heterogeneity, the random effects model was used to calculate the overall effect size. Statistical analysis was performed using CMA v.2.0 software and *P* value less than 5% was considered as a significant level.

### Publication bias

To test the Publication bias, Egger’s regression test was used. Moreover, *P* value <0.1 was considered as a sign of publication bias. In the Egger’s regression test, the mean changes in troponin levels in the two groups (sevoflurane and isoflurane) were considered 24 hours after entering the ICU.

### Additional analysis

#### Subgroup analysis and sensitivity analysis

In the studies included in this meta-analysis, the troponin level in types I and T was reported. Sub-groups were performed based on the type of troponin in both groups of sevoflurane and isoflurane in patients undergoing cardiac surgery. Sensitivity analysis was performed by removing the studies in which the level of troponin in types I and T was reported in children.

## Results

### Search results

**Figure 1:**
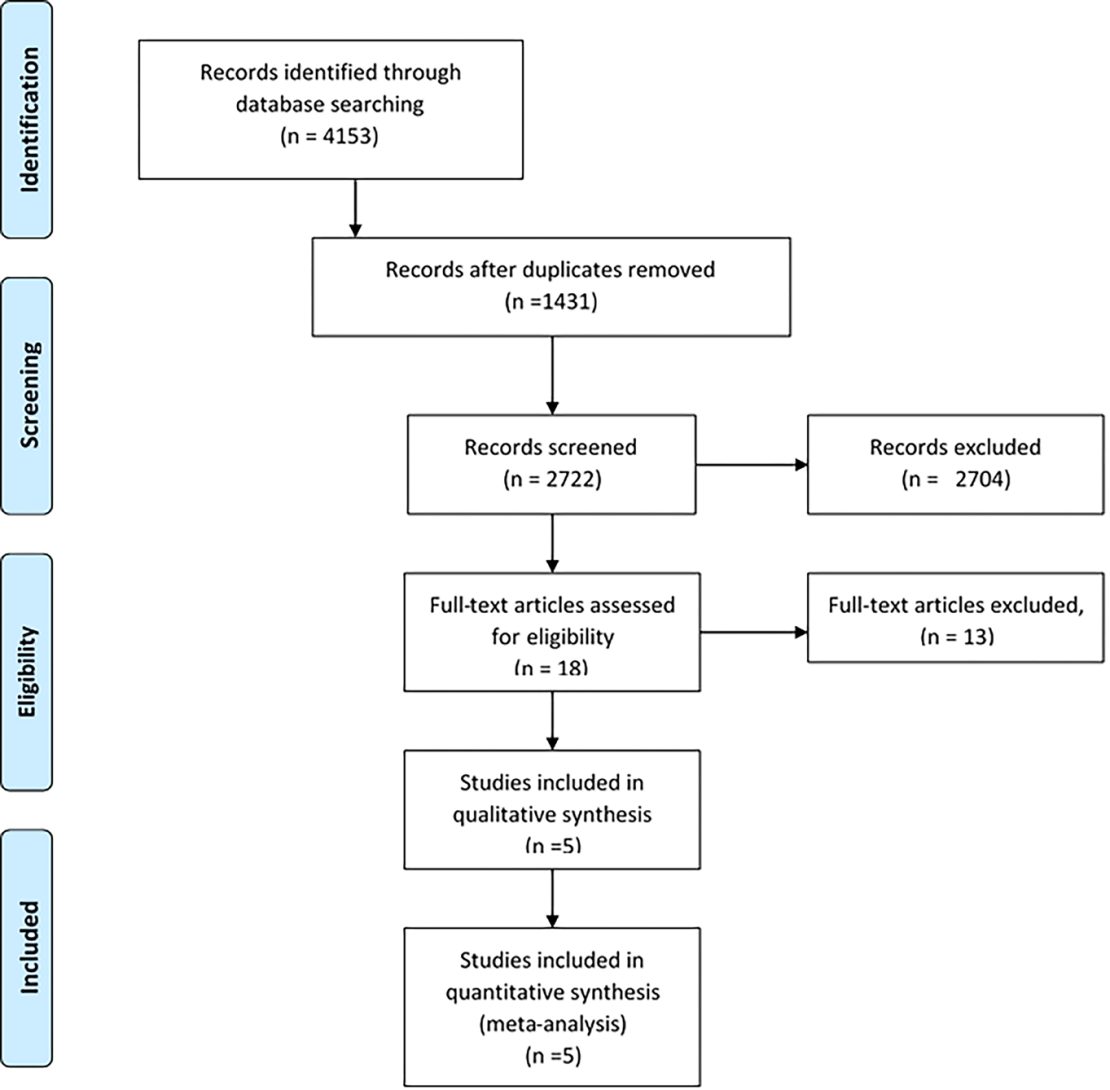
PRISMA flow chart for studies included in the review.

In the systematic search performed in databases, 4153 articles were identified. 1431 duplicate articles were recorded, and 2704 articles were excluded after reviewing their titles and abstracts. After reviewing the full texts of the articles, 13papers were excluded from the study. Finally, 5 eligible studies were included for the meta-analysis.^10,11,20,26,27^ The PRISMA flow diagram for clinical trials is shown in Figure 1.

### Study characteristics

The final 5 selected studies had been published between 2007 and 2017. A total of 381 participants in our meta-analysis (190 in the sevoflurane group and 191 in the isoflurane group) were anesthetized. One study had been conducted on children^11^ and four other studies had been conducted on adults.^10,20,26,27^ The mean age of children in the sevoflurane and isoflurane groups were 3.07 and 3.47 respectively. Moreover, the mean age of adults in sevoflurane and isoflurane groups were 60.16 and 59.89 respectively. All studies assessed troponin within 48 hours after surgery. Furthermore, two studies measured cardiac troponin cTnT^26,27^ and three studies measured cardiac troponin cTnI.^10,11,20^ The characteristics of the included studies are shown in Table 1.

**Table 1:** Characteristics of the included studies

**First Author**	**Year**	**Surgical type**	**Type of study**	**Number of cases (sevoflurane/isoflurane, n)**	**Group of age**	**Troponin**	**Outcome of studies**
Soliman	2017	CABG	Prospective randomized	114/114 228	Adult	Troponin I, ng/mL	Troponin I and creatine kinase-MB (CKMB)].
Hassan	2015	CPB	Prospective randomized	30/30	Child 2 and 6 years	Troponin I, ng/mL	Measuring cardiac troponin I (cTnI)
Ceyhan	2011	CABG	Prospective randomized	20/20	Adult	Troponin T, ng/mL	Measuring cardiac troponin -T, creatine kinase (CK) and CK-MB
Yildirim	2009	CABG	Randomly	20/20	Adult	Troponin I, ng/mL	Measuring cardiac troponin I (cTnI)
Delphin	2007	OPCAB	Prospectively	6/7	Adult	Troponin T, ng/mL	Troponin enzyme levels for isoflurane and sevoflurane

CABG: coronary artery bypass grafting surgery, CPB: Cardiopulmonary bypass, OPCAB: off-pump coronary artery bypass.

### Quality of evidence (GRADE)

In this study, changes in cardiac troponin levels isoflurane and sevoflurane anesthesia was investigated 7 times. Form evidence obtained from the meta-analysis, this is the grading place during these seven time periods. Based on the GRADE approach, the power of evidence were in a low level at the time of ICU, 6, 12, 48 hours and (Baseline=At time 24 h (ICU)), in moderate level at the time 48 ours (Baseline = arrival in ICU), and in high level at the time 24 ours. The result of GRAD is shown in Table 2.

**Table 2:** Grades of the evidence for comparing cardiac troponin levels using sevoflurane and isoflurane in patients undergoing cardiac surgery

**Subgroup**	**No. of study**	**Limitations**	**Inconsistency**	**Indirectness**	**Imprecision**	**Publication bias**	**Strength**	
At time ICU	3	No serious	Serious	No serious	Serious	Detected	-3.63 (-4.64,-2.62)	Low
6 h	3	No serious	Serious	No serious	Serious	Undetected	-0.05 (-1.2,1.1)	Low
12 h	2	No serious	Serious	No serious	Serious	-	-0.36 (-2.16,1.45)	Low
24 h	4	No serious	Serious	No serious	No serious	Undetected	1.19 (0.59,1.79)	High
48 h	2	No serious	Serious	No serious	Serious	-	0.36 (-1.57,2.29)	Low
24 h (Basline=At time ICU)	4	No serious	Serious	No serious	Serious	Undetected	0.23 (-0.01,0.47)	Low
48 h (Basline=At time ICU)	3	No serious	Serious	No serious	Serious	Undetected	0.67 (0.43,0.9)	Moderate

### Risk of bias

**Figure 2:**
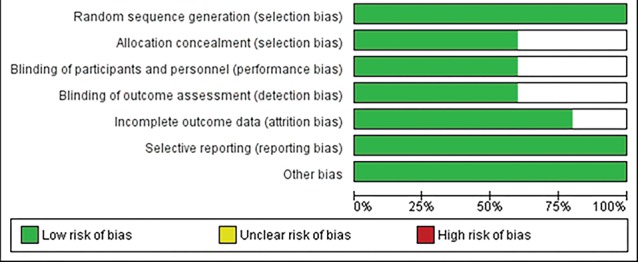
Risk of bias and applicability concerns graph for comparing cardiac troponin levels using sevoflurane and isoflurane in patients undergoing cardiac surgery.

**Figure 3:**
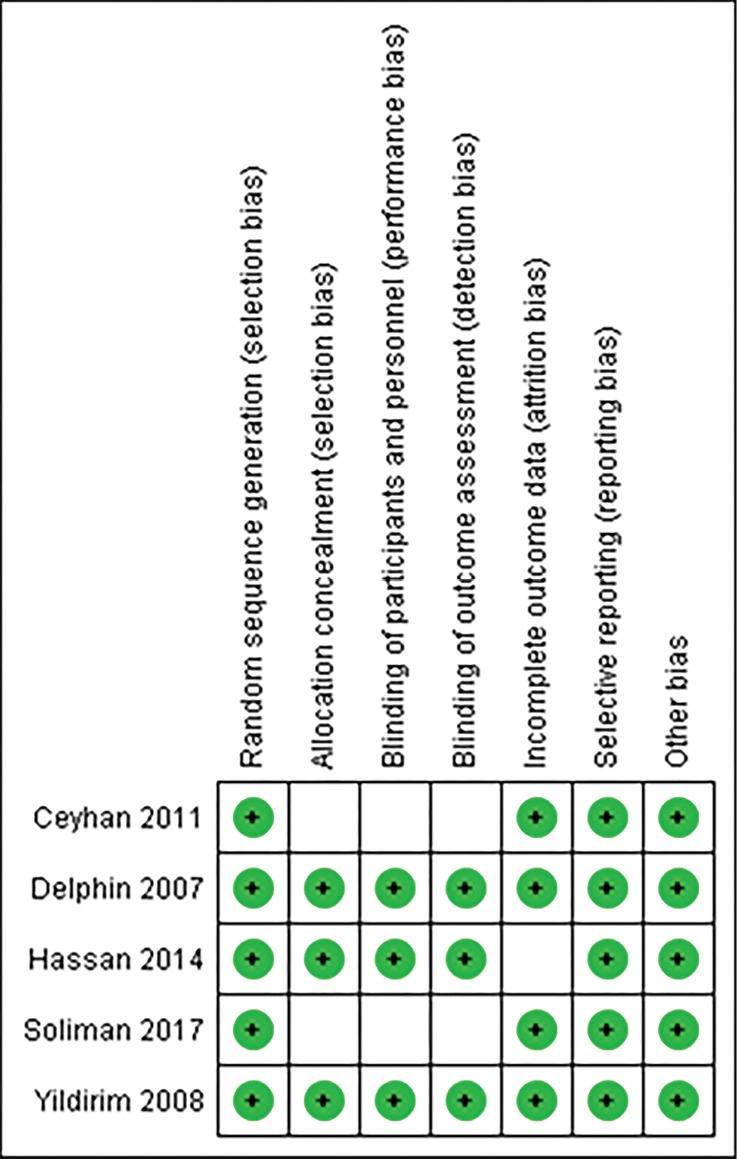
A summary table of review authors’ ratings of risk of bias and applicability concerns for each study of comparing cardiac troponin levels using sevoflurane and isoflurane in patients undergoing cardiac surgery.

Two studies in this meta-analysis had not provided explanations about the concealment of patient staff and outcome assessors; therefore, they might have been influenced by selection performance and detection bias.^10,26^ In one of the studies , whilst there was no explanation for the Attrition sample, there might have been an attrition bias.^12^ Only one study had sufficient explanations about blinding outcome assessor and the rest of the studies might have been affected by the detection bias^20^ (Figure 2 and 3).

### Meta-analysis results

Meta-analysis results from comparing cardiac troponin levels using sevoflurane and Isoflurane in patients undergoing cardiac surgery: arrival in ICU, 6, 12, 24, and 48 hours after ICU admission.

### Baseline = before surgery

#### Arrival in ICU

**Figure 4:**
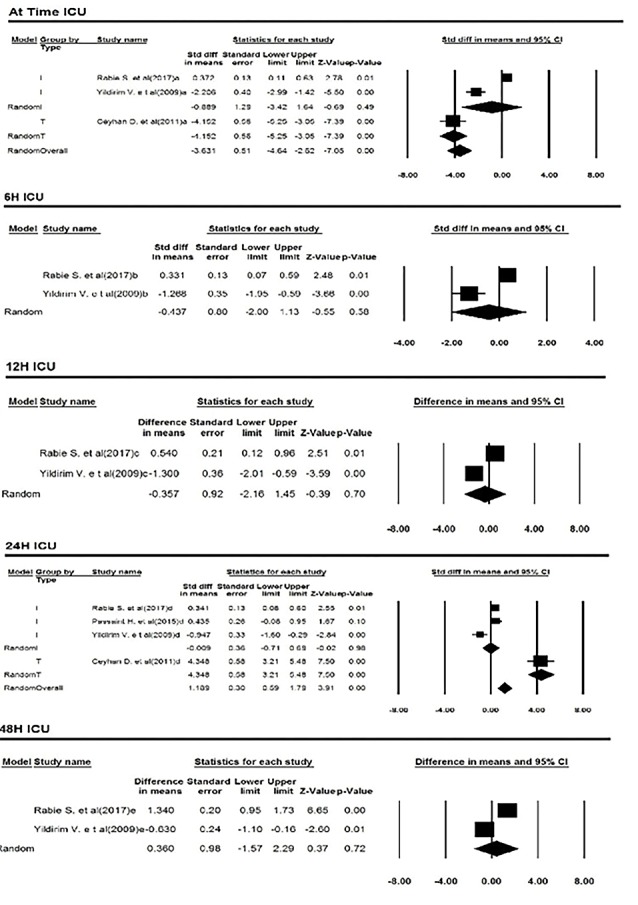
Forests plot mean difference between cardiac troponin levels using sevoflurane and isoflurane in at time ICU, 6 hour, 12hour, 24hour and 48 hour after ICU (baseline =At time ICU).

Three studies reported troponin levels at the time of admission to the ICU. Two of studies had reported the cardiac troponin I and one of the studies had reported the cardiac troponin T. The homogeneity between the studies was significant (Q= 92.09, *df*= 2, *P* < 0.001, I^2^= 97.83). Based on meta-analysis, troponin levels in patients with anesthesia group of isoflurane were 3.63 ng/mL less than those in the sevoflurane anesthetic group. This difference was statistically significant. (MD= -3.63; 95% CI= -4.64 to -2.62; *P* < 0.001). A forest plot is available in Figure 4.

#### Six hours after ICU admission

Three studies reported cardiac troponin6 hours after entering the ICU. In these studies, cardiac troponin I had been investigated. The heterogeneity among these studies was not significant (Q=22.86, *df*=2, *P* <0.001, I^2^=91.25(. Based on meta-analysis, changes in cardiac troponin level in anesthetized patients with isoflurane were 0.05 ng/mL less than those anesthetized with anesthetized sevoflurane. This difference was not statistically significant (MD = -0.05; 95% CI= -1.20 to 1.10; *P* value=0.93). A forest plot is available in Figure 4.

#### Twelve hours after ICU admission

Two studies reported cardiac troponin 12 hours after entering the ICU. In these studies, cardiac troponin I was studied. This difference was statistically significant (Q=19.11; *df*=1; *P* < 0.001; I^2^=94.77). Based on meta-analysis, troponin levels were 0.36 ng/mL less in patients anesthetized with anesthetized isoflurane than those given sevoflurane with anesthesia. This difference was not statistically significant (MD= -0.36; 95% CI= -2.16 to 1.45; *P* value=0.93). A forest plot is available in Figure 4.

#### Twenty-four hours after ICU admission

Four studies reported cardiac troponin levels within 24 hours of entering the ICU. In these studies, 3 studies about cardiac troponin I were investigated, and the difference was statistically significant Q=13.73; *df*=3; *P* < 0.001; I^2^=95.21(. Based on meta-analysis, troponin levels were 1.19 ng/mL more in patients anesthetized with anesthetized isoflurane than those given sevoflurane with anesthesia. This difference was statistically significant (MD = 1.19; 95% CI = 0.59 to 1.79; *P* < 0.001). A forest plot is available in Figure 4.

#### Forty-eight hours after ICU admission

Two studies reported cardiac troponin levels within 48 hours of entering the ICU. In these studies, cardiac troponin I was investigated. The heterogeneity among these studies was not significant (Q = 39.09; *df* = 1; *P* < 0.001; I^2^ = 97.44). Based on meta-analysis, cardiac troponin levels were 0.36 ng/mL more in patients treated with anesthetized isoflurane than those given sevoflurane with anesthesia. This difference was not statistically significant (MD = 0.36; 95% CI = -1.57 to 2.29; *P* = 0.72). A forest plot is available in Figure 4.

#### Comparison of troponin level changes between two groups (baseline =at time ICU)

#### Twenty-four hours (Baseline = arrival in ICU)

**Figure 5:**
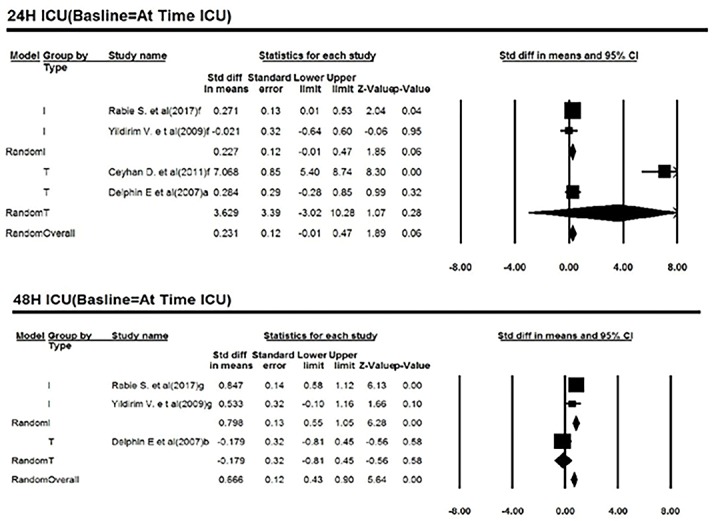
Forest plot mean difference between cardiac troponin levels using sevoflurane and isoflurane 12 hour and 48 hour after ICU.

Four studies reported cardiac troponin levels within 24 hours of entering the ICU. Two of studies had reported the cardiac troponin I and two studies had reported cardiac troponin T. The heterogeneity among these studies was not significant (Q = 64.07; *df* = 3; *P* < 0.001; I^2^ = 95.32). Based on meta-analysis, cardiac troponin levels were 0.23 ng/mL more in patients treated with anesthetized isoflurane than those given sevoflurane with anesthesia. This difference was not statistically significant (MD = 0.23; 95% CI = -0.01 to 0.47; *P* = 0.06). A forest plot is available in Figure 5.

#### Forty-eight hours (baseline = at time ICU)

Three studies reported cardiac troponin levels within 48 hours of entering the ICU. Two of studies have reported the cardiac troponin I and one of the cardiac troponin T. The homogeneity between the studies was not significant (Q = 8.82; *df*=2; *P* = 0.01; I^2^ = 77.33) Based on meta-analysis, changes in troponin levels were 0.67 ng/mL more than in patients treated with an isoflurane-based drug than those treated with anesthetized sevoflurane. This difference was not statistically significant. )MD = 0.67; 95% CI = 0.43 to 0.90; *P* < 0.001). A forest plot is available in Figure 5.

### Sensitivity analysis and subgroup analysis

Sensitivity analysis was performed by removing one of studies.^11^ After removing Hassan et al study which had been conducted on children, troponin levels in isoflurane group in 6 hours was 0.44 units less than in sevoflurane group (MD = -0.44; 95% CI = -2.00 to 1.13; *P* = 0.58). It also changes after 24 hours, the isoflurane group was 2.28 times higher than the sevoflurane group (MD = 2.28; 95% CI = 1.43 to 3.12; *P* < 0.001). In Table 3 and online resources 2 and 3 (Supplementary file 1), results of sensitivity analysis are showed. In Table 4, results of subgroup analysis are showed.

**Table 3:** Sensitivity analysis for comparing cardiac troponin levels using sevoflurane and isoflurane in patients undergoing cardiac surgery

**Time**	**Groups**	**Effect size and 95% confidence interval**				**Test of null (2-tail)**		**Heterogeneity**			
		**Number Studies**	**Point estimate**	**Lower limit**	**Upper limit**	**Z-value**	***P*** **value**	**Q-value**	**df (Q)**	***P*** **value**	**I** ^ 2 ^
6 h	I	2	-0.44	-2.00	1.13	-0.55	0.58	18.55	1	0.00	94.61
24 h	I	2	-0.27	-1.53	0.99	-0.42	0.68	12.84	1	0.00	92.21
	T	1	4.35	3.21	5.48	7.50	0.00	0.00	0	1.00	0.00
	Overall	3	2.28	1.43	3.12	5.29	0.00	62.65	2	0.00	96.81

**Table 4:** Subgroup analysis for comparing cardiac troponin levels using sevoflurane and isoflurane in patients undergoing cardiac surgery

**Time**	**Type of troponin**	**Number studies**	**Effect size and 95% CI**				**Test of null (2-Tail)**		**Heterogeneity**		
			**Mean difference**	**Lower limit**	**Upper limit**	***Z*** **value**	***P*** **value**	**Q-value**	**df (Q)**	***P*** **value**	**I** ^2 ^
At time ICU	I	2	-0.89	-3.42	1.64	-0.69	0.49	37.20	1	0.00	97.31
	T	1	-4.15	-5.25	-3.05	-7.39	0.00	0.00	0	1.00	0.00
	Overall	3	-3.63	-4.64	-2.62	-7.05	0.00	92.09	2	0.00	97.83
6 H	I	3	-0.05	-1.20	1.10	-0.09	0.93	22.86	2	0.00	91.25
12 H	I	2	-0.36	-2.16	1.45	-0.39	0.70	19.11	1	0.00	94.77
24 H	I	3	-0.01	-0.71	0.69	-0.02	0.98	13.73	2	0.00	85.43
	T	1	4.35	3.21	5.48	7.50	0.00	0.00	0	1.00	0.00
	Overall	4	1.19	0.59	1.79	3.91	0.00	13.73	2	0.00	95.21
48 H	I	2	0.36	-1.57	2.29	0.37	0.72	39.09	1	0.00	97.44
24 H (Baseline = at time ICU)	I	2	0.23	-0.01	0.47	1.85	0.06	0.72	1	0.40	0.00
	T	2	3.63	-3.02	10.28	1.07	0.28	57.02	1	0.00	98.25
	Overall	4	0.23	-0.01	0.47	1.89	0.06	64.07	3	0.00	95.32
**48 H (Baseline = at time ICU)**	I	2	0.80	0.55	1.05	6.28	0.00	0.80	1	0.37	0.00
	T	1	-0.18	-0.81	0.45	-0.56	0.58	0.00	0	1.00	0.00
	Overall	3	0.67	0.43	0.90	5.64	0.00	8.82	2	0.01	77.33

### Publication bias

The base result of Egger regression test publication bias between studies in this meta-analysis was not significant (t-value = 0.63; *df* = 2; *P* = 0.59).

## Discussion

With the increased prevalence of cardiovascular disease, millions of people are undergoing cardiac surgery.^2,29^ Cardiac troponin (T and I) are of biomarkers for identification of myocardial injuries in patients undergoing cardiac surgery. High level of troponin is an index for this diagnosis.^29-31^ Volatile anesthetic, sevoflurane, and isoflurane in patients undergoing heart surgery creates hemodynamic stability and maintain anesthesia depth. Additionally, the properties of these drugs in preventing perfusion and the protective effect on myocardium have led to their use in heart surgery.^19,32^ Inhalational anesthetics such as sevoflurane have neuroprotective effects and protective effects on hepatocytes as well.^33-35^

Myocardial preconditioning during anesthesia is a cellular protective method in which exposure to a volatile anesthetic reduces cardiomyocyte injury. Preconditioning by sevoflurane has displayed cardiac protective effects on hypoxia/reoxygenation injury, but the related mechanism is uncertain. In a study syntax in 1A (STX1A), an essential regulator in cardiac disease was considered to be the target gene of microRNA-34a-5p.^36^

The myocardial protective effect of sevoflurane may be related to the enhancement of mitochondrial respiratory function of cardiomyocyte after upregulation of HIF-1α gene expression.^37^

This meta-analysis was performed with 5 studies in 381 patients undergoing cardiac surgery for the evaluation of cardiac troponin during the use of anesthetics of isoflurane and sevoflurane. The results of this study showed changes in levels of troponin in patients entering the ICU. The level of troponin in patients who were under anesthesia with isoflurane increased less than the level of troponin in patients who were with anesthetics sevoflurane. Based on the subgroup analysis results, level of troponin T and I in the patient under cardiac surgery with isoflurane was less. The mean difference in troponin level between the two groups of isoflurane and sevoflurane was not significant 6 hours, 12 hours, and 48 hours after entering the ICU. However, sevoflurane had a better effect on troponin level 24 hours after surgery. However, according to the results of subgroup analysis, sevoflurane and isoflurane, respectively, had better effects on troponin T and I.

In our study, the mean difference of troponin levels was also calculated using sevoflurane and isoflurane 24 and 48 hours after surgery, as compared to the time in ICU. Within 24 hours after surgery, the troponin level of sevoflurane was lower. However, there was no significant difference between the two drugs. Also, for troponin T and I, the level of sevoflurane was lower than isoflurane. In subgroup analysis, there was no difference in the level of troponin T and I between the two drugs. 48 hours after the surgery, the increased troponin levels were lower with sevoflurane compared with isoflurane. In subgroup analysis, based on the type of troponin, sevoflurane had a better effect on the type of troponin I and isoflurane had a better effect on the type of troponin T.

Overall, the results of the GRADE showed that our studies of strength had moderate evidence. Therefore, perhaps the cause of the difference between the results of troponin with isoflurane and sevoflurane at the times of this meta-analysis was the differences in the type of operation as well as the different hemodynamic conditions of patients undergoing cardiac surgery. In the past, some studies on inhaled anesthetic agents were performed to investigate their effects on myocardial protection.^38-40^ Studies have also been conducted on troponin levels in patients undergoing cardiac surgery. Moreover, in all of these studies, there were contradictions similar to our study. Some reported that there was no difference between the two drugs, while others reported superiority of one over the other. For example in the study conducted by Parker et al^19^ and Bennett et al,^41^ it was shown the protective effect in sevoflurane and isoflurane was similar and no changes were observed in troponin levels using the two drugs. Two studies^28,42^ reported that sevoflurane provides better hemodynamic and protective conditions in the heart.

In the study by Venkatesh et al^18^ which was performed to compare the sevoflurane and isoflurane, sevoflurane had a better protective effect on the heart. Sevoflurane had a better effect on reducing the amount of troponin T in 24 hours after surgery. In the study conducted by Passaint et al,^11^ in 30 children aged 2 to 6 years, troponin I in the isoflurane group was higher than that of sevoflurane. Sensitivity analysis was 6 and 24 hours after ICU. When we removed Passaint et al^11^ study from the analysis, there was a small change in the amount of pooled relative to when we entered this research.

As we found in the present study, differences and similarities in our research results are consistent with previous studies. However, in general, isoflurane and sevoflurane do not have a significant negative effect on troponin levels. Moreover, in the two previous meta analytic studies which had focused on the comparison of isoflurane and sevoflurane with propofol, it has been shown that inhalation anesthetics such as isoflurane and sevoflurane have an acceptable protective effect on myocardial infarction in comparison with propofol.^1,39^ In this meta-analysis, we encountered a few limitations such as the small number of clinical trials and low sample size. Moreover, some of the studies had not reported troponin levels in each group.

## Conclusion

This meta-analysis showed that blood troponin levels were significantly lower Arrival in ICU with isoflurane and 24 hours after ICU with sevoflurane in patients who underwent cardiac surgery. Generally, given the small mean difference between isoflurane and sevoflurane, it seems that isoflurane and sevoflurane do not have a negative effect on cardiac troponin level.

## Competing interests

None.

## Ethical approval

The Ethics panel of the School of Health Management and Information Science Iran University of Medical Sciences indicated that ethics approval was not required for this systematic review.

## Acknowledgments

This study was funded by the School of Health Management and Information Science Iran University of Medical Sciences (code: 691). School of Health Management and Information Science did not involve in study design; in the collection, analysis and interpretation of data; in the writing of the report; and in the decision to submit the article for publication.

## Supplementary Materials


Supplementary file 1 contains Search Strategy For Systematic Literature Review And Forest Plot Figures For Sensitivity Analysis.
