# Supplementary material for: Comparing cardiac troponin levels using sevoflurane and isoflurane in patients undergoing cardiac surgery: a systematic review and meta-analysis
Source: J Cardiovasc Thorac Res. 2020 Feb 12;12(1):1–9. doi: 10.34172/jcvtr.2020.01 (PMC7080340; doi:10.34172/jcvtr.2020.01)
Supplement: Supplementary file 1 — contains Search Strategy For Systematic Literature Review And Forest Plot Figures For Sensitivity Analysis. [file jcvtr-12-1-s001.pdf]

Supplementary file 1

Online Resource 1. Search Strategy for Systematic Literature Review in Pubmed   DATE: February 30th, 2019

#1	Heart surgery[MeSH] OR heart operation or cardiac surgery, valve replacement[MeSH] or Coronary artery bypass surgery[MeSH]
#2	Extracorporeal Circulation[MeSH] OR Circulation, Extracorporeal OR Circulations, Extracorporeal OR Extracorporeal Circulations
#3	Cardiopulmonary Bypass[MeSH] OR Bypass, Cardiopulmonary OR Bypasses, Cardiopulmonary OR Cardiopulmonary Bypasses
#4	Heart-Lung Bypass[MeSH] OR Bypass, Heart-Lung OR Bypasses, Heart-Lung OR Heart Lung Bypass OR Heart-Lung Bypasses
#5	Anesthesia OR volatiles anesthetics OR Anesthesias OR inhalational Anesthesia
#6	#1 OR #2 OR #3 OR #4 OR #5
#7	Isoflurane [supplementary concept]OR Forane OR Terrell OR Isofluran*
#8	Sevoflurane [supplementary concept] OR sevofluran* OR sevorane OR ultane
#9	# 6 And # 7 And #8

6H ICU

Model	Study name	Std diff in means	Standard error	Lower limit	Upper limit	Z-Value	p-Value
	Rabie S. et al(2017)b	0.331	0.13	0.07	0.59	2.48	0.01
	Yildirim V. e t al(2009)b	-1.268	0.35	-1.95	-0.59	-3.66	0.00
Random		-0.437	0.80	-2.00	1.13	-0.55	0.58

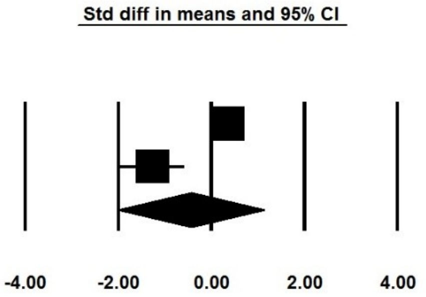

Online Resource 2. Forest plot sensitivity analysis between cardiac troponin levels using sevoflurane and Isoflurane 6 h ICU.

24H ICU

Model	Group by Type	Study name	Std diff in means	Standard error	Lower limit	Upper limit	Z-Value	p-Value
	I	Rabie S. et al(2017)d	0.341	0.13	0.08	0.60	2.55	0.01
	I	Yildirim V. e t al(2009)d	-0.947	0.33	-1.60	-0.29	-2.84	0.00
Random	I		-0.267	0.64	-1.53	0.99	-0.42	0.68
	T	Ceyhan D. et al(2011)d	4.348	0.58	3.21	5.48	7.50	0.00
Random	T		4.348	0.58	3.21	5.48	7.50	0.00
Random	Overall		2.277	0.43	1.43	3.12	5.29	0.00

(Statistics for each study)

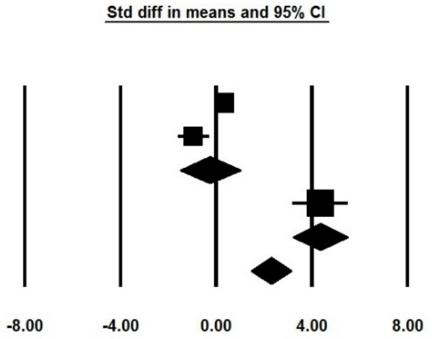

Online Resource 3. Forest plot sensitivity analysis between cardiac troponin levels using sevoflurane and isoflurane 24 h ICU
